# Behçet uveitis: Current practice and future perspectives

**DOI:** 10.3389/fmed.2022.968345

**Published:** 2022-09-07

**Authors:** Shereen H. Aboul Naga, Lameece Moustafa Hassan, Radwa T. El Zanaty, Mohammad Refaat, Rana H. Amin, Gaafar Ragab, Mahmoud M. Soliman

**Affiliations:** ^1^Ophthalmology Department, Faculty of Medicine, Cairo University Hospital, Uveitis Subspecialty Clinic, Giza, Egypt; ^2^Rheumatology and Clinical Immunology Unit, Department of Internal Medicine, Faculty of Medicine, Cairo University, Giza, Egypt

**Keywords:** Behçet's disease, uveitis, diagnostic criteria, ocular investigation, immunosuppression, biologics, COVID-19

## Abstract

Described as early as Hippocrates in his “Third Book of Endemic Diseases,” Behçet's Disease (BD), also known as “The Silk Road Disease” following its initial demographics, consists of a triad of recurrent oro-genital ulcers and associated uveitis. Current demographics and rising percentages of patients seen far beyond the Silk Road in Ocular Inflammatory Disease and Uveitis Clinics list BD uveitis as one of the frontliners of non-infectious autoinflammatory eye diseases. Clinical features of BD and juvenile-onset BD are detailed alongside various approaches in classification and suggested algorithms for diagnosis that are outlined in this review. With the ongoing Human Microbiome Project and studies such as the MAMBA study, the role of the human microbiome in BD is highlighted in the pathophysiology of BD to include the current research and literature perspective. Furthermore, with the advancement of recent diagnostic and investigative techniques, especially in the field of Optical Coherence Tomography (OCT), disease-related characteristics are updated to encompass SD, EDI and OCT-angiography characteristics of BD. Having entered the era of biologic therapy, the role of various specific cytokine-blocking biologic drugs, such as TNF-α inhibitors (e.g., adalimumab, infliximab), interferon α-2a inhibitors, IL-6 and IL-1 inhibitors are presented and contrasted alongside the conventional immunosuppressant drugs and the classic old gold standard: corticosteroids (systemic or local). Finally, with the ongoing SARS-CoV-2 pandemic, it was not possible to conclude the review without reviewing the latest evidence-based literature reporting BD morbidity in this era, the observed pattern and treatment recommendations as well as those related to reported post-vaccine complications and emergence of BD.

## Introduction

Historically, “The Silk Road Disease”, now better known as Behçet's Disease, has been described as early as Hippocrates in his “Third Book of Endemic Diseases” (1–3). However, the clinical trial of recurrent oro-genital ulcers and associated ocular uveitis remained obscure until the dermatologist Hulusi Behçet defined it as a syndrome, having seen it in three native patients of Middle Eastern origin in 1937 (3). Today, Behçet Disease has extended beyond its localities along the “Ancient Silk Road” to encompass a more global reach and is expanding further north and south (3, 4).

Today, the development of international registries dedicated to specific or rare autoimmune disease entities provides a powerful, structured multidisciplinary tool for data collection, disease identification, epidemiological studies on more current, evidence-based and multi-centric basis. One of these registries is the AIDA International Registry for BD patients, which is considered a successful model and is currently being developed and implemented for other diseases (5).

BD is a multi-system disease. The most frequent clinical features manifest at a mucocutaneous and ocular level. However, cardiovascular, articular, gastrointestinal as well as neurological manifestations frequently accompany or even precede the disease, making diagnosis more difficult (4, 6–12). Given that BD remains a clinically diagnosed entity and its heterogeneous nature of presentation, criteria for BD were developed and continue to be refined and re-evaluated to allow for the ethnic variabilities encountered across various demographic ethnicities (9, 13, 14).

In a recent epidemiologic study by Abdelwareth et al., data for 313 uveitis patients managed at the Uveitis Subspecialty Clinic of Kasr Al Aini, Cairo University Hospital (the largest tertiary referral center in Egypt) between May 2015 and May 2017 was statistically examined. Out of the 313 patients, 75.4% were diagnosed having a specific etiology, with Behçet uveitis at the lead, constituting 29.1% of the clinic's patient profile for that time period (6). Hassan et al. further analyzed the cohort of non-infectious uveitis patients in multiple Egyptian tertiary health care centers (Cairo, Tanta and Benha University Hospitals), identifying BD as the leading diagnostic entity (51.2%) (7).

In this review article, the authors introduce and highlight the latest updates over the past decade, regarding diagnosis and management of Behçet disease and its associated uveitis. However, they will remark on the juvenile-onset BD (Jo-BD), which presents a real challenge due to the difficulty in diagnosis and management of this less common subgroup.

## Pathogenesis

HLA-B51 has been confirmed as the principal genetic predisposing factor by Genome-wide Association studies (GWAS). A positive test increases the risk of developing BD by 5.79-fold (10, 11). This genetic predisposition, together with associations discovered by the GWAS to other non-HLA genes (10), in addition to evidence of altered microbiome especially gut in Behçet patients and infectious agents such as Streptococcus sanguinis (isolated from the oral mucosa of patients with Behçet's disease), enter into an interplay, that triggers a sustained immune response. This disrupts a previously intact T-cell homeostatic environment and results in a state of chronic inflammation in these individuals (10, 12–14). The new understanding of these immuno-pathogenic processes have expanded the standard treatment protocols, which now include the more recent biologic therapy, especially TNF-alpha antagonists, which are administered for control of the ongoing and repeated disrupted immune response (12).

The IL-23/IL-17 axis plays an important role in immune mediated pathologies, including uveitis Increased levels of IL-23 trigger the maturation of pathogenic Th17 cells (rather than the homeostatic subtype). These Th17 cells in turn promote the production of proinflammatory cytokines via the JAK/STAT signaling cascade. Furthermore, IL-23 continues to upregulate its receptor expression, thus stabilizing a proinflammatory response environment, aggravating the inflammatory response (15, 16).

The microbiome is defined as the genetic material of all microorganisms (bacteria, fungi, protozoa and/ or viruses) living both on the surface and inside the human body. The majority inhabit the large intestine and help regulate important body functions as food digestion, blood coagulation and vitamin production. Consequently, this microbiome is mappable. such as by the Human Microbiome Project (HMP) sponsored by the National Human Genome Research Institute (NHGRI) and part of the National Institutes of Health (NIH) in the United States (17).

The suggested hypothesis is that an alteration or disturbance of a susceptible individual's microbiome by other pathogenic microorganisms can trigger a cascade process altering his/her genetic material which may ultimately translate into the expression of various autoimmune or autoinflammatory diseases, e.g., multiple sclerosis, diabetes, and currently Behçet's disease.

Currently, the MAMBA Study is an ongoing randomized, cross-over, open trial assessing the effect of regional variations and nutritional modification on a patient's gut microbiome and its possible outcome on BD (8).

The underlying pathology of BD is that of a relapsing-remitting vasculitis of vessels of all sizes, affecting multiple organ systems and manifesting in a gamut of heterogeneous clinical signs (8, 9). While defined as a non-infectious auto-inflammatory disease, theories of an underlying infectious agent date back to Hulusi Behçet, in a trial to explain the recurrent pattern and nature of the oral ulcerations. However, all failed to isolate a viral pathogen. Currently, isolating streptococcal strains from the extraocular lesions in BD patients, still suggests a possible association to an infectious triggering agent. However, the theory remains controversial.

## Updated diagnostic criteria

Since its description in 1937, 18 sets of diagnostic or classification criteria have been developed for BD (18). The most famous of which was published in 1990 by the International Study Group “ISG” in a collaboration of 7 countries to bring a consensus on one set of criteria (12).

Despite its high specificity, subsequent application and evaluation of the “ISG” criteria in individual countries repeatedly lacked diagnostic sensitivity relative to other criteria that had been proposed and were not included in the classification (18, 19). It also did not allow for variations in the symptoms of BD, incomplete expression, and failed to discriminate BD from the separate entity of inflammatory bowel diseases (18, 20). Thus, the International Team for the Revision of the International Criteria for BD (ITR-ICBD) was formed under the auspices of the Epidemiology Research Group of the International Society for Behçet's Disease (Table 1). The aim of this team was to re-assess the sensitivity and specificity of existing criteria sets, including ISG, on a large cohort of patients from 27 countries, in order to create a new evidence-based scheme with good discriminatory properties regardless of patient ethnicity (14). It is noteworthy that the ICBD performed better in an Egyptian cohort of cases when compared with that of the ISG (21).

**Table 1:** International criteria for Behçet's disease—point score system: scoring > 4 indicates Behçet's diagnosis (14).

***Sign/Symptom***	***Points***
Ocular lesions	2
Genital aphthosis	2
Oral aphthosis	2
Skin lesions	1
Neurological symptoms	1
Vascular manifestations	1
+ve Pathergy test	^*^1^*^

* Pathergy test is optional and the primary scoring system does not include pathergy testing. However, where pathergy testing is conducted one extra point may be assigned for a positive result.

Despite the availability of multiple criteria sets for diagnosing the presence or absence of the disease, none currently determine the “probability” of Behçet diagnosis when put in a list of differentials (22).

Another classification is worth noting, the Japanese criteria set, which was defined by the Japanese Ministry of Health in 1987 (23, 24). Despite predating the aforementioned classifications, it clearly shows the role demographic and environmental criteria play on the phenotypic expression of BD (Table 2). Over the past 30 years, some studies suggest that a new phenotype of BD has evolved in Japan and Korea, where the majority of patients are presenting with incomplete Behçet's and milder phenotypes. This was in comparison to the 80s, where BD was identified as the leading cause of non-infectious uveitis in Japanese patients, a statistic that has shifted recently in favor of sarcoidosis as the principal cause (24–27).

**Table 2:** A comparison between the Japanese, ISG, and ICBD criteria for diagnosis of BD.

	***ICBD Scoring System***	***ISG Scoring System***	***Japanese Scoring System***
*Oral ulcer*	2 points	Mandatory	Major criterion
*Genital ulcer*	2 points	Minor criterion	Major criterion
*Skin region*	1 point	Minor criterion	Major criterion
*Uveitis*	2 points	Minor criterion	Major criterion
*Pathergy test*	1 point	Minor criterion	Not included
*Arthritis*	Not included	Not included	Minor criterion
*Epididymitis*	Not included	Not included	Minor criterion
*GIT*	Not included	Not included	Minor criterion
*Neurological*	1 point	Not included	Minor criterion
*Vascular*	1 point	Not included	Minor criterion

The Japanese criteria require 3 major criteria, or uveitis and 1 major or 2 minor criteria. ISG requires 3 out of 5 components and the ICBD diagnoses BD at ≥ 4 points (24). Adapted from Kirino and Nakajima (24).

Colours highlight corresponding impact of criteria in the different scoring systems (major is similar to a score of 2 or mandatory for example).

The Japanese criteria is of significance, as they take into account the higher incidence of gastrointestinal Behçet's (12%) vs. the markedly lower Mediterranean as well as Western incidence of (1–7%). On the other hand, pathergy is rarely positive in Japanese patients and hence omitted entirely from this classification set (24). A patient diagnosed with intestinal, neurological or vascular BD is classified as a special-BD subtype, and noticeably, these patients advance faster in their disease.

## Ocular Behçet's clinical presentations

Just as the main disease, ocular Behçet may present with various pictures and degrees of severity in 50 to 70% of patients. It may initially begin unilaterally. However, it is usually a bilateral disease and the second eye soon follows. The usual age of onset is around 30 years of age and is often more severe in the male patients. Behçet's uveitis is recurrent, non-granulomatous, and extends from the anterior to the posterior pole. It is a progressive sight-threatening disease that may involve parts or the entire uveal tract and may blind up to 25% of patients within a course of 10 years, after which disease progression tends to stabilize (28, 29). Thus, good disease control is essential within this window to save the eye either from the direct ocular manifestations of Behçet's uveitis or its potentially and equally blinding complications (30–32).

**Figure 1:**
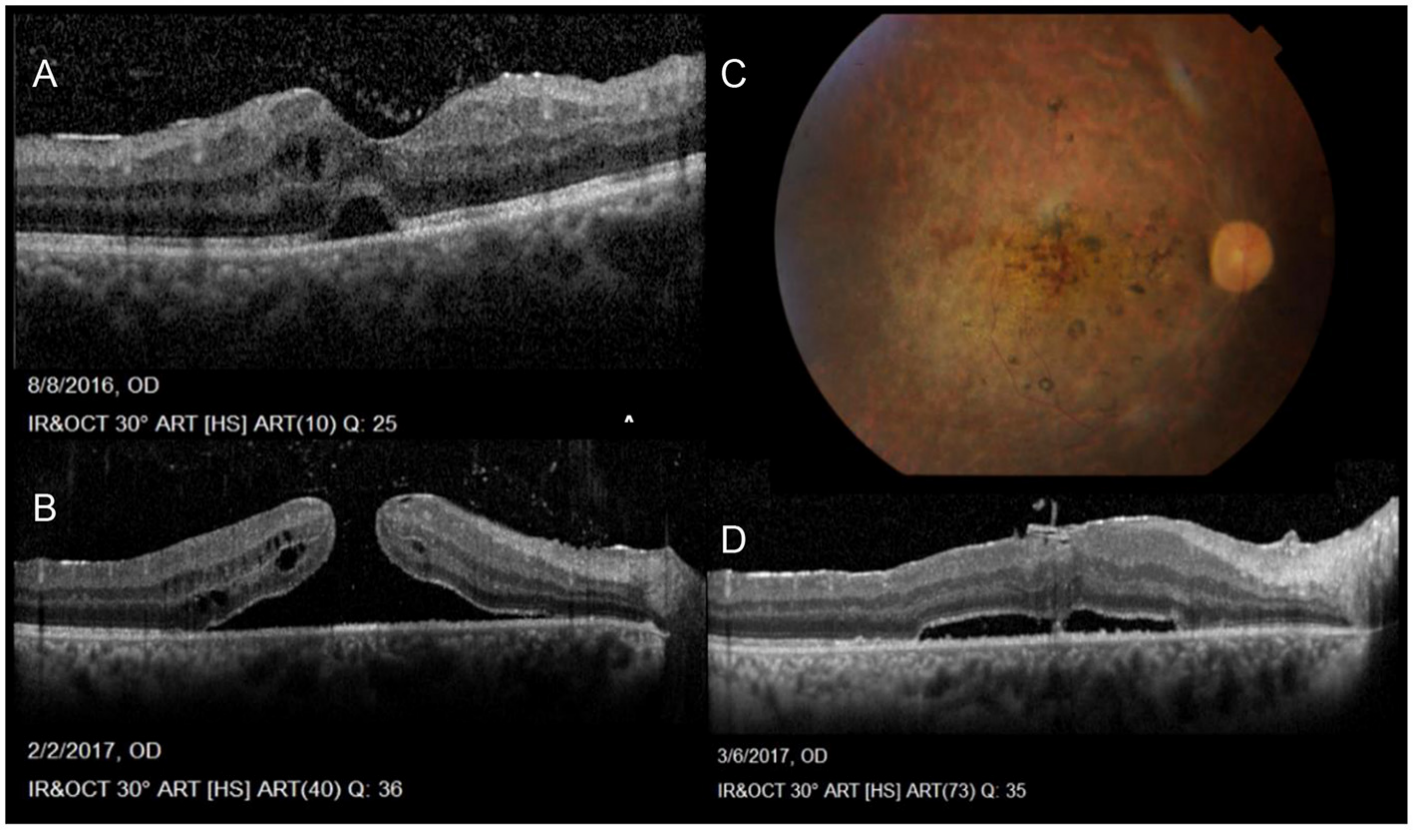
Sequelae of Behçet Uveitis: **(A)** Cystoid macular edema, epiretinal membrane, neurosensory detachment and vitreous opacities denoting vitritis **(B)** Progression of the cystoid changes in to a full thickness, macular hole **(C)** Color photos Post vitrectomy sealing of the hole, also showing severely attenuated vessels, pale discs and laser marks **(D)** OCT post PPV showing residual NSD following vitrectomy with peeling of ILM and sealing of the hole (Series courtesy of Dr. Soliman MM, MD).

**Figure 2:**
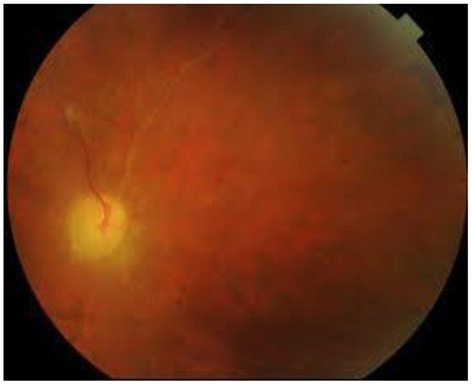
Colored fundus photo of Behçet uveitis showing a pale disc and ghost vessels following the occlusive vasculitis (Image courtesy of Dr. Soliman MM, MD).

Tugal-Tutkun et al. reported anterior uveitis in 11% of cases, posterior uveitis in 28.8%, while panuveitis involvement was seen in 60.2% of their entire cohort of 880 patients (1,567 eyes). Intermediate uveitis in the form of isolated vitritis without anterior or posterior involvement (clinically and angiographically) was also reported more often in early rather than late onset BD (31–33). However, the latest SUN classification criteria published in 2021 does not include isolated vitritis in its diagnostic criteria, rather in association with anterior, posterior or panuveitis (34). On the other hand, vitritis accompanying posterior segment involvement is common and may be so dense, obscuring the fundus view. Retinal vasculitis, predominantly periphlebitis, but also combined with arteritis are a main feature and are often accompanied by vaso-occlusive retinopathy with retinal and vitreous hemorrhages, retinal ischemia, neovascularization and secondary neovascular glaucoma (**Figure 2**) (35). Papillitis is also seen as part of the vasculitis typical of BD, while neovascularization at the disc is rare and may be secondary to chronic, uncontrolled inflammation but not ischemia (35, 36). All through, macular edema is a leading complication, often a blinding sequelae of posterior uveitis (37). Macular holes have also been reported with BU and associated changes involving the vitreo-macular interface (Figure 1) (35, 38, 39).

Isolated anterior uveitis is rare. Fine dusting of the endothelium accompanies iritis and the typical shifting hypopyon may form. The hypopyon invariably points to involvement of the posterior segment. Throughout an attack, the eye may appear white or show strong ciliary injection (28, 31, 37). Finally, Behçet patients may also present with complications of the disease due to its chronic relapsing remittent nature, such as cataract, synechiae and glaucoma as well as the above mentioned posterior segment complications. Untreated, the eye will show the end-stage appearance of an ischemic, thinned out retina, with sheathed ghost vessels and optic atrophy (35, 40) (Figure 2).

In 2020, Tugal-Tutkun et al. published an algorithm for the diagnosis of BD uveitis based on characteristic ocular findings. Their study consisted of 4 steps: (i) survey of expert opinion on characteristic features of ocular involvement in BD; (ii) retrospective clinical data collection and analysis; (iii) prospective clinical data collection; (iv) development of a diagnostic algorithm (41). The variables identified to provide the highest accuracy for the diagnosis of BD uveitis, which constitutes an estimated 15% of cases, included the presence of superficial retinal infiltrates or related sequelae, RNFL defects, angiographic signs of occlusive retinal vasculitis and diffuse retinal capillary leakage in the absence of granulomatous anterior uveitis or choroiditis in patients with vitritis. The authors postulated that a combination of these ocular findings, rather than individual BU-associated lesions would be more readily recognizable. Accordingly, the presence of all the afore-mentioned signs (criteria) in a patient would then suggest the highest (92%) probability of a BU diagnosis (42). This algorithm however requires further validation in larger, multicentric studies and larger clinical cohorts.

## Pediatric Behçet's disease

Pediatric Behçet is a rare and difficult condition to diagnose. It includes children up to the age of 16 years and its pattern differs from adults in appearance and predominance of principal diagnostic signs (43, 44). Terminology further differentiates between pediatric BD, which fully manifests before the age of 16 years and juvenile-onset BD (JO-BD), which presents with a childhood onset of the disease but does not fulfill the criteria (18). The percentage of JO-BD is reportedly between 4 and 26% of Behçet patients. Not only the paucity, but also the latency of complete disease manifestation and the heterogeneous presentations pose a diagnostic, as well as treatment challenge in the younger age groups (4, 18, 44, 45).

Attempts at improving classification and diagnostic criteria for Behçet's disease are not limited to adults and continue to attempt to bypass regional variabilities of clinical expression, such as the skin pathergy test, which is not applicable to all demographics (14). From 18 classification sets of BD, mainly 2 are in use for adult BD, the ISG and the ICBD classification, while only one, the Pediatric Behçet Disease (PEDBD) consensus, which was published in 2015 addresses pediatric BD separately (Table 3) (18, 46).

**Table 3:** Consensus classification of pediatric Behçet's disease (46).

***Item***	***Description***	***Value/item***
Recurrent oral aphthosis	≥3 attacks per year	1
Genital ulceration/aphthosis	Typically with scar	1
Skin involvement	Necrotic folliculitis, acneiform lesions, erythema nodosum	1
Ocular involvement	Anterior uveitis, posterior uveitis, retinal vasculitis	1
Neurological signs	With exception of isolated headaches	1
Vascular signs	Venous thrombosis, arterial thrombosis, arterial aneurysm	1
Three of six items are required to classify a patient as having pediatric Behçet's Disease.		

Koné-Paut et al., suggested a revised consensus based on a large cohort study of 219 patients from 42 centers located in 12 different countries. The ethnic subgroups were about one third European-Caucasian, one third North African and one third Middle Eastern-Caucasian (46). Their findings were tested regarding confirmed (156 patients) and unconfirmed (63 patients) against the ISG Criteria for BD as well as the ICBD classifications. On the other hand 410 patients with 3 different disease entities distinct from BD were provided from the Eurofever Database as negative controls to test for the validity of the identified diagnostic criteria (12, 14, 46).

Similar to adults, the most common presenting sign and often the first at a mean age of 8 to 9 years is recurrent, widespread multiple or single oral ulcers (44, 47, 48), with Sota et al. deriving similar data from the AIDA Registry network (49). Genital ulcers are comparatively less frequent than in adults, however they are the second most common presenting sign in children and are seen predominantly in females. Unlike their oral counterparts, they are characterized by a tendency to scar. Chronologically, with a longer latent period between the first and second presenting sign compared to adults, oro-genital ulceration is often followed, at a mean age of 10 to 13 years, by skin lesions, neurological symptoms and musculoskeletal manifestations) (18, 45, 46).

Regarding the frequency of ocular involvement, Atmaca et al. and Krause et al., reported a similar ocular involvement rate between adults and children (50, 51). Koné-Paut et al., on the other hand suggested a lower prevalence of ocular involvement in childhood BD. However, the presence of ocular signs, such as anterior and/or posterior uveitis or retinitis have a higher morbidity and carry a worse prognosis compared to adults (46). Uveitis was reportedly more common in boys often running a severe course (47, 52), and according to Koné-Paut et al., bilateral involvement was mostly noted in the European-Caucasian cohort of their series (4, 45, 46).

## Ocular investigations in Behçet's disease

The complexity in the diagnosis of BD lies in the fact that there is no specific diagnostic test. Alone, a positive pathergy test or positive typing for HLA-B51 are not diagnostic. Rather, the diagnosis is based on the cumulation of multiple clinical signs that fall within the aforementioned diagnostic criteria (29).

Cases of BU, especially those with posterior segment involvement, often require ocular imaging. Currently, multimodal imaging is heavily relied upon, not only in the diagnosis of this condition, but also in assessment of disease activity, outlining as well and monitoring response to treatment (53).

### Color photography

Although not new, fundus photography is a simple, economic but often overlooked tool. It can document the grade of vitreous haze for disease monitoring and can document the transient nature of retinal infiltrates, which is particular to BU (40, 54, 55).

### Indocyanine green angiography (ICGA)

Although BD is a systemic vasculitis, vasculitis and inflammatory lesions are mainly documented at the level of the retina (sparing the choroidal vessels). Thus, ICGA may be used to differentiate Behçet's disease from other entities primarily affecting the choroid, while lacking any specific or pathognomonic diagnostic signs for BD itself (56, 57).

### Fundus fluorescein angiography (FFA)

**Figure 3:**
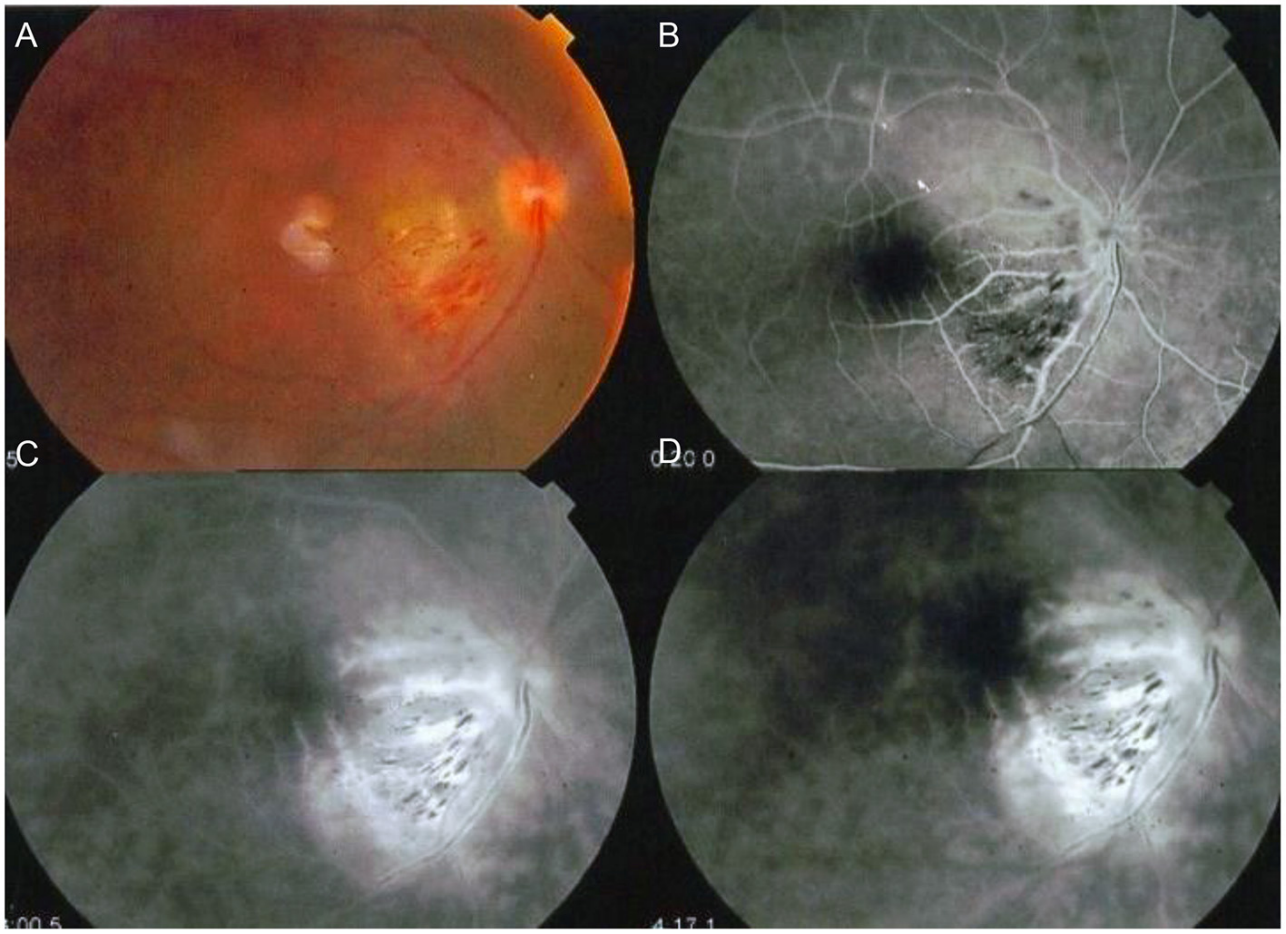
FFA of a patient with Behçet s vasculitis. **(A)** Color photo showing macular branch retinal vein occlusion and disc edema. **(B–D)** FFA images showing the macular vein occlusion and widespread vasculitis with characteristic “fern-like configuration” (Images courtesy of Dr. Soliman MM, MD).

Even though there have been rapid advances in ocular imaging techniques, FFA remains the gold standard investigation for diagnosis and follow-up of the characteristic occlusive vasculitis or active (leaking) vasculitis seen in Behçet's posterior uveitis (55) (Figure 3).

**Figure 4:**
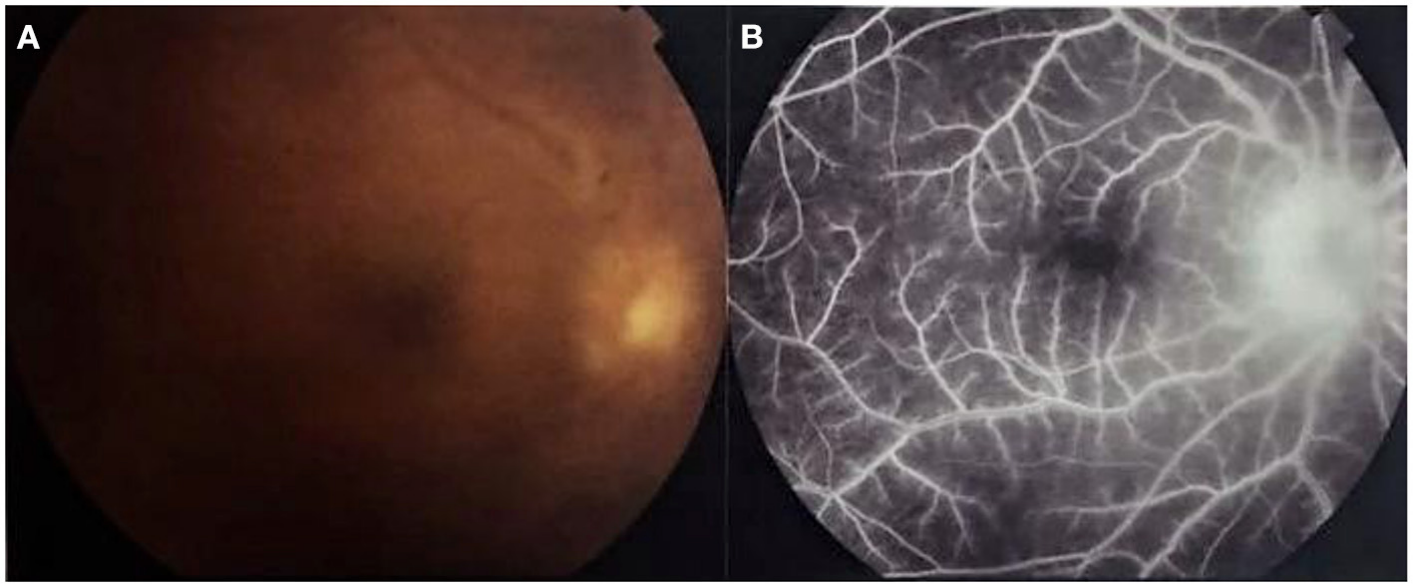
Color photo and fundus fluorescein angiography (FFA) of a patient with active Behçet uveitis. **(A)** Color photo showing disc edema, sheathed vessels, blunt macular reflex. **(B)** FFA showing active vasculitis (fern-like configuration typically extending beyond one quadrant) and disc leakage (Images courtesy of Dr. Wassef A, MSc.).

Ozdal et al. reported that the most common FFA findings of posterior segment involvement of ocular BD were vasculitis in 38% of eyes, optic disc edema in 14.8% and macular edema in 11.3% (40). The most characteristic FFA finding in BU is a “fern-like capillary leakage” that indicates activity. Although similar vasculitis may be observed in other uveitic entities, in BD, the leakage often involves more than three quadrants of the fundus (55) (Figure 4).

In a study on 23 eyes with inactive ocular BD, FFA imaging detected uveitic activity in 52.1% of the studied eyes. This was observed in the form of vasculitis (30%), macular edema (17.3%), macular ischemia (8.6%) and peripheral occlusive vasculitis (4.3%) (58). This finding suggests that inflammation remains radiologically active despite clinical uveitic quiescence and may indicate that the current treatment is inadequate (55).

The introduction of the more recent ultra-wide fluorescein angiography (UWFA) has allowed the visualization of vasculitis anterior to the equator in BD, which can cause peripheral leakage, ischemia, and neovascularization, that are otherwise difficult to detect clinically. In a 2014 study, UWFA imaging of 33 eyes unmasked peripheral vasculitis in 28 eyes (84.8%) and peripheral retinal non-perfusion in 22 eyes (66.7%), which were not clinically evident. Subsequently, immune-modulatory treatment was modified based on the UWFA findings in 13 of 20 patients (65%) (59).

### Optical coherence tomography

#### Spectral domain OCT

In eyes with suitable optical media, optical coherence tomography (OCT) provides a rapid and non-invasive means of investigating macular complications, the most frequent being cystoid macular edema, which should be closely monitored by OCT (53).

Other studies have demonstrated that decreased foveal thickness and disruption of the photoreceptor inner and outer segment junction detected by OCT are associated with poor visual function, indicating irreversible damage to the macula (60).

The appearance of retinal infiltrates denotes an activation of intraocular inflammation in the posterior segment. Spectral Domain OCT (SD-OCT) sections through retinal infiltrates typically show focal retinal thickening, increased hyper-reflectivity and back shadowing, which resolve without visible chorioretinal scarring (53). Oray et al., observed localized retinal nerve fiber layer (RNFL) defects as sequelae of superficial retinal infiltrates affecting the posterior pole in patients with BU. They proposed these OCT findings could serve as an early indicator of posterior pole involvement (61).

Recently, OCT has also been used to objectively measure the associated degree of vitreous inflammation in BU, as a tool for monitoring activity. Behçet neuroretinitis often reveals itself with a localized vitreous condensation overlying the infiltrated optic disc. Optically, OCT scans through the optic disc may show a “smoking volcano” picture or a “mushroom-shaped cloud that caps the plume” corresponding to the clinical finding. Thus, OCT allows non-invasive monitoring of the disc infiltration and overlying inflammatory reaction (62, 63).

#### Enhanced depth OCT

***Enhanced Depth Imaging (EDI)***, the recent addition to most OCT devices, has allowed histologic in-depth examination of the choroid. There are multiple studies investigating choroidal thickness by this EDI mode of SD-OCT in patients with BU.

**Figure 5:**
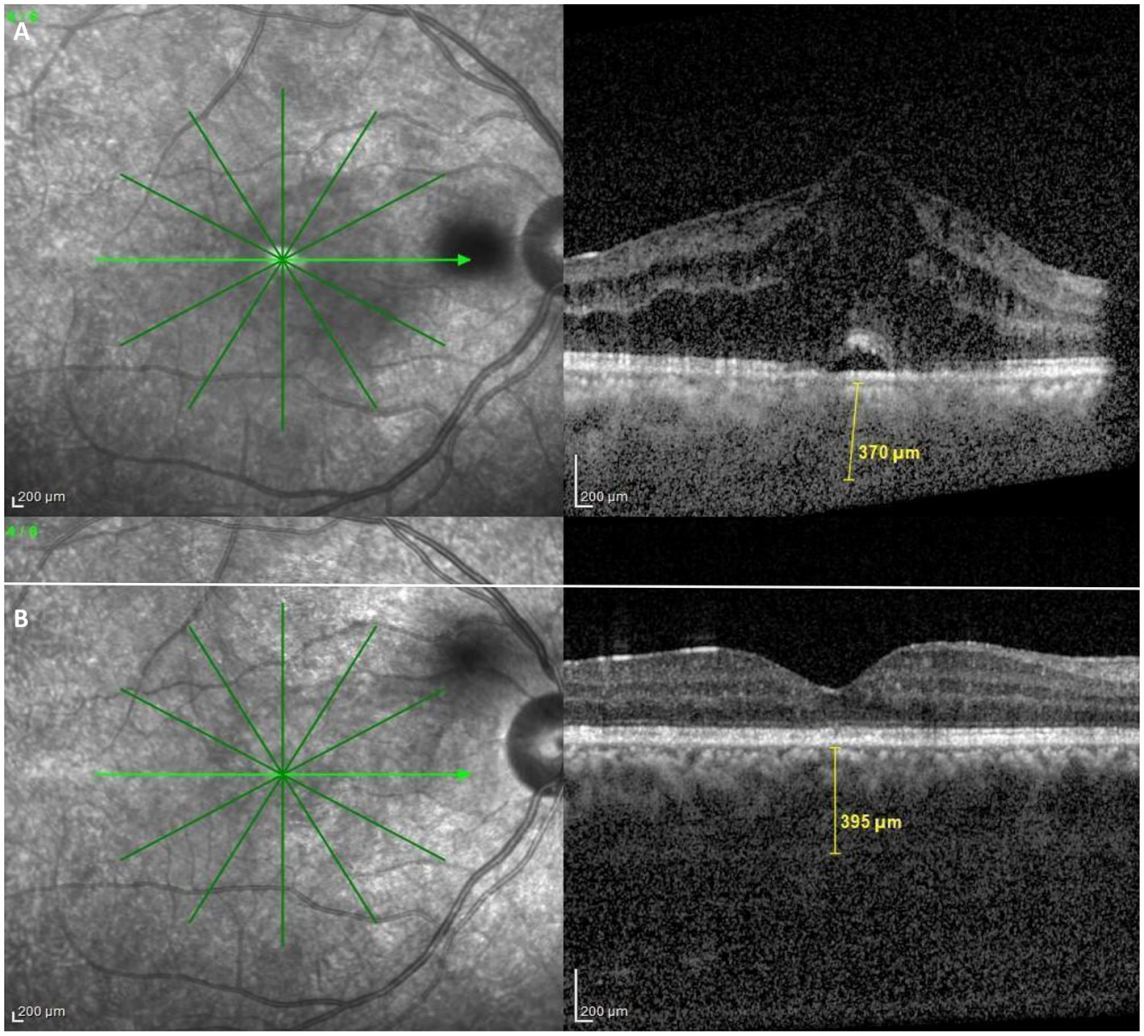
OCT findings in Behçet Uveitis. **(A)** OCT of an active BU patient showing center involving cystoid macular edema, neurosensory detachment, epiretinal membrane and increased subfoveal choroidal thickness. **(B)** OCT of an inactive BU patient showing diffuse parafoveal edema, epiretinal membrane and also above average subfoveal choroidal thickness (Images courtesy of Dr. Wassef A, MSc.).

Kim et al., studied choroidal thickness during active and quiescent BU. They observed choroidal thickening during the active phase. Furthermore, subfoveal choroidal thickness during the quiescent phase remained significantly greater than that of normal subjects (Figure 5). They also found that the degree of reduction in choroidal thickening significantly correlated with an improvement in retinal vascular leakage on FFA (64). In support of these findings, longitudinal follow-up data by Ishikawa et al. also suggested a decrease in choroidal thickness with resolution of intraocular inflammation. However, according to their study, this change did not translate into any significant corresponding visual improvement (65).

#### OCT angiography

***Optical coherence tomography angiography (OCTA)*** is a novel imaging technique that resolves and displays high-resolution, depth-resolved, en face images of the retinal and choroidal microvasculature by calculating motion contrast in OCT B-scans acquired repeatedly at the same location (66).

**Figure 6:**
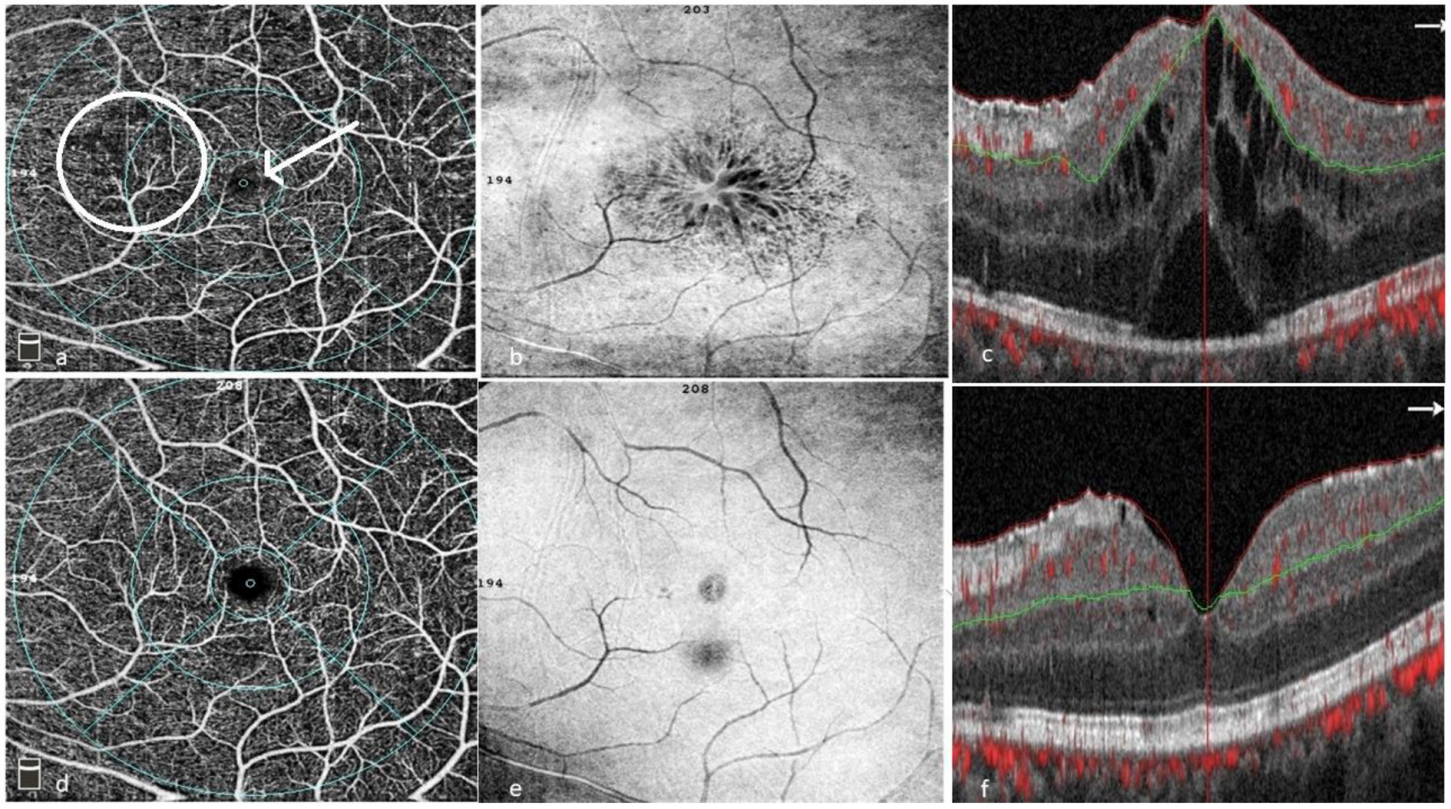
Patient with BU pre- **(a–c)** and post treatment **(d–f)**. **(a)** OCTA of the superficial capillary plexus showing areas of capillary drop outs, capillary telangiectasia, disorganization and hyporeflective areas corresponding to the cystoid spaces on SD-OCT. **(b)** En face OCT showing cystoid spaces involving the fovea. **(c)** SD-OCT with center involving cystoid spaces, subfoveal neurosensory detachment and a hyperreflective epiretinal membrane. **(d)** OCTA showing resolution of most capillary changes (telangiectasia, drop outs, and disorganization). **(e)** En face showing resolution of cystoid changes with residual epiretinal membrane. **(f)** SD-OCT showing resolution of cystoid changes and neurosensory detachment with residual diffuse edema and an epiretinal membrane (Images courtesy of Dr. Wassef A, MSc.).

In 2016, Khairallah et al., demonstrated that the main changes detected by OCTA were retinal capillary non-perfusion, rarefied, dilated or shunting perifoveal capillary vessels, disorganization of the normal architecture of the capillary network, enlargement of FAZ, and reduction of capillary vessel density (CVD) (Figure 6). They determined that the deep capillary plexus (DCP) was more affected than the superficial capillary plexus (SCP) (67).

Numerous studies have been conducted to assess microvascular changes associated with BD. In an Egyptian study done on 22 eyes with BU during activity and following remission, the authors proposed that OCTA can be used to monitor activity of Behçet's posterior uveitis. The superficial capillary plexus (SCP) density was more sensitive to the activity status. On the other hand, the deep plexus (DCP) and the FAZ area -being areas where damage is more irreversible- were more useful as prognostic indicators (68).

Somkijrungroj et al., proposed that deep capillary affection in BU occurs at an early stage of the disease and proceeds regardless of the activity status of the disease. They suggested that it correlates positively with the number of reported attacks, thus there tends to be a bigger irreversible component of the hypoperfusion in the deep plexus than in the superficial (69). Likewise, Accorinti et al. found that even in inactive stages of the disease, a permanent alteration of the macular microvasculature might be observed and that the duration of a disease-free period was strictly related to OCTA findings, indicating that in inactive uveitis, the vessel density is inversely related to the number of ocular relapses and cannot be restored over time (70).

OCTA may be superior to FFA for visualizing, characterizing, and quantifying perifoveal microvascular alterations in active BU. OCTA images allow clear vessel visualization, due to the absence of dye leakage phenomenon, seen on FFA (67). However, FFA still remains indispensable, as there is no correlation between the presence of peripheral retinal ischemia on FFA and any of the OCTA pathologic features. Thus, FFA remains, currently, the only means for detecting and evaluating peripheral retinal capillary non-perfusion and neovascularization and is better at showing retinal vascular and optic disk leakage, which are definite signs of activity in BU (67).

Possibly, with the advent of the wide-field OCTA imaging, more peripheral retinal data can be obtained that may supplement ultra-wide field FFA imaging and do so in a non-invasive, dye-free technique. Currently the drawback lies in the trade off in resolution for the large acquisition area over a short time (71).

## Updates in treatment

Medical management of the BU should be tailored according to the mode of presentation (anterior, posterior or panuveitis), as well as the severity of the attack, as there are no standard rules of treatment (72). The European League Against Rheumatism (EULAR) published first guidelines for management of the disease in 2008 (73). Over the past decade, additional numerous studies were published addressing different therapies which lead to the development of updated EULAR guidelines in 2018 (13). The consensus in uveitis management has clearly shifted to being a multidisciplinary collaboration between experienced uveitis specialists and rheumatologists. Another recommendation was the limitation of steroid administration to short-term and acute stage control, to be replaced by DMARDs or biologic therapy according to EULAR and American Academy of Ophthalmologists' guidelines. Furthermore, the American Academy of Ophthalmology recommended bypassing the “classic DMARDs” in favor of anti-TNF-alpha agents in severe, sight-threatening uveitis (13). In this section we review the updates on the different systemic drugs used in the management of BD-associated uveitis.

### Steroids

The 2018, the EULAR updated guidelines recommended administering glucocorticoids in posterior segment ocular BD patients, but only in combination with steroid-sparing therapies such as azathioprine (AZA), cyclosporine A (CsA), interferon alpha or monoclonal anti-TNF antibodies. The role of systemic steroids was defined to primarily address an acute episode, to control the attack and prevent extensive tissue damage (13). In cases with severe vitritis, extensive occlusive retinal vasculitis, retinitis and optic neuropathy, high doses of steroids (whether pulse methylprednisolone regimen followed by oral prednisone 1 mg/kg/day, or directly skipping to the latter) are given, bearing in mind steroid-related systemic complications (7). Tapering steroids, in addition to steroid-sparing therapy, are then initiated targeting maintenance of remission (13, 37).

When BD manifests as isolated anterior uveitis, usually topical steroids and cycloplegics are sufficient to control the disease (74), yet a manifestation in the form of an aggressive attack with hypopyon necessitates systemic steroids, especially when associated with poor prognostic factors, such as young age and male gender (13, 32).

Regional steroids, in the form of sub-Tenon Triamcinolone acetonide (TAA) injections are also effective in controlling active ocular disease and are often used in conjunction with other systemic treatment regimens in severe cases. Adjunct intravitreal steroid administration has also been reported to control ocular inflammation and macular edema in ocular Behçet, administered either as TAA intravitreal injections or, more recently, in the form of fluocinolone acetonide or dexamethasone implants, especially in cases with refractory CME. Success was reported both anatomically as well as visually and may require the management of complications such as the temporary rise of intraocular pressure and/or cataract formation. These complications were reportedly higher with fluocinolone acetonide vs. dexamethasone implants (75–77).

### Steroid-sparing immunosuppressants

This drug class is used to allow for steroids withdrawal while controlling the disease activity and reducing or preventing relapses. The choice of the drug(s) as well as the doses should be done in collaboration with an expert rheumatologist for drug monitoring.

Currently, immunosuppressant therapy for BD uveitis can be grossly divided into conventional treatment AZA and the biologic agents such as TNF-alpha inhibitors and Interferon alpha-2a (78–80).

#### Conventional treatment (CT)

Randomized controlled trials (RCT) have proven that the antimetabolite AZA and T-cell inhibitor CsA to be effective in the treatment of posterior uveitis in BD as well as in successfully decreasing the frequency of relapses (81–84). These evidence-based results maintained their validity and thus the updated 2018 EULAR guidelines recommended the use of these two drugs in the initial therapy of posterior uveitis. On the other hand, mycophenolate mofetil and cyclophosphamide were not included in the latest EULAR guidelines update (13). Once control of inflammation on low-dose maintenance steroids ( ≤ 5–7.5 mg/day) is achieved for several months, a progressive tapering of the immunosuppressant dose is begun. Generally reducing the dose by 10% every 2 to 3 months until discontinuation, which may be achieved after 18 to 24 months of treatment. However, a longer duration of immunosuppressant medication is often necessary.

**Azathioprine (AZA)** is one of the two most commonly used conventional treatment drugs in the control of systemic BD, and specifically in Behçet's uveitis. It requires 2–3 months to achieve full effect. During this period, control of the active disease should be achieved with steroids. The dose of AZA usually used is 2.5 mg/kg/day with a maximum of 3 mg/kg/day and has proven efficacious in BD uveitis, improving the visual acuity, reducing relapses and halting progression into severe disease (14).

In spite of necessary regular monitoring of blood picture and liver enzymes, AZA is generally considered a well-tolerated drug. A trial of tapering and withdrawal can be initiated after a period of remission and may extend beyond 18–24 months (79, 80).

**Cyclosporine A (CsA)** is the second most commonly used conventional drug and is usually started at 2–5 mg/kg/day in two divided doses which can be increased gradually until good control is achieved in addition to the low oral steroids dose. Similar to AZA, CsA has proven to be effective in improving visual acuity and reducing severity of the attacks with fewer recurrences (84). The main side effects of CsA are nephrotoxicity and hypertension (85). Due to its neurotoxicity, it is contraindicated in cases with neuro-Behçet's (86). After disease control is achieved, the drug is to be tapered very gradually over a long period like AZA to prevent rebound inflammation. The concomitant use of AZA and CsA, whether as first or second-line therapy, has shown efficacy in controlling ocular BD with periodic monitoring of systemic side effects (14, 73).

#### Biologics

While still some of the most commonly used CT drugs have been associated with refractory BU cases or treatment side effects. Their use as first-line therapies has decreased since the emergence of biologics. Due to their potent and fast effects, biologics are now used alone or in combination therapy in refractory ocular Behçet's cases or sometimes even as first line treatment in severe sight-threatening attacks (13).

### Tumor necrosis-alpha (TNF-alpha) inhibitors

In BD, TNF-alpha production by macrophages, CD4+ and CD8+ T-cells, and Natural Killer cells is increased (87, 88). The reduction of circulating TNF-alpha by blocking agents has resulted in dramatic improvement in disease activity as demonstrated in many trials especially in those with severe pan- or posterior uveitis.

Anti-TNF-alpha drugs used are recombinant monoclonal antibodies directed against TNF-alpha. Pre-treatment protocol with biologics necessitates the exclusion of tuberculosis and hepatitis B or C as well as occult malignancies before starting therapy due to possible flare-ups of these diseases by the drugs. Multiple effective and inter-changeable agents are currently present, should one drug option fail (89). Usually an additional dose of an immunosuppressant is necessary with some of the TNF-alpha blockers to prevent anti-chimeric, or anti-human, antibody production, which decreases the drug's efficacy resulting in secondary failure (90–92).

**Adalimumab (ADA)** is a fully human monoclonal antibody directed against TNF-alpha. It is one of the few drugs that has been tested in RCTs against a placebo, in both active and quiescent non-infectious uveitis (VISUAL I and VISUAL II studies, respectively) (93, 94), in which Ocular BD represented 7% of the uveitic cases enrolled. Due to its superiority over placebo in improving central retinal thickness and control of disease activity (but not in terms of macular edema), the European Medicines Agency (EMA) and the US Food and Drug Administration (FDA) approved ADA for non-infectious non-anterior uveitis in 2016. Adalimumab is administered via a subcutaneous injection at an adult dose of 40 mg every 2 weeks.

Numerous uncontrolled studies, such as the data presented by Fabiani et al. and Urruticoechea-Arana et al. also showed significant results regarding efficacy of ADA in improving BD uveitis (95–97). Not only was it superior to placebo in the control of disease activity but also a higher percentage of patients on ADA were able to withdraw oral steroids (94).

Adalimumab has also been tried in the pediatric BD subgroup where early initiation of the drug in two children succeeded in control of the disease activity with tapering of topical and systemic steroids and hence avoiding complications (98).

Humira is the reference adalimumab drug investigated in all of the above trials. Several biosimilars-adalimumab (bio-ADA) are still under investigation regarding their efficacy in ocular BD. A very recent study by Soheilian et al. reported the significantly positive results achieved by bio-ADA in improving visual acuity, decreasing vitreous haze and improving anterior chamber activity in 48 patients with refractory BU on conventional treatment (99). Sota et al. report good results in controlling retinal vasculitis and disease activity while preserving visual acuity (100, 101).

**Infliximab (IFX)** is another TNF-alpha blocker in the form of a chimeric monoclonal antibody. It is usually reserved for refractory cases or used as a first-liner in case of severe posterior uveitis with higher risk of tissue damage or visual loss. Infliximab is administered at a dose of 3 to 5 mg/kg in a slow intravenous infusion over 2–3 h. Loading regimen includes repeating the dose at the 2nd week, then the 6th week, then every 6–8 weeks for maintenance of disease control (102).

Many trials have demonstrated the rapid, profound effect infliximab had on BD uveitis. The drug has resulted in rapid remission of the disease and improved visual acuity. It also reduced the number and severity of attacks in comparison with other immunosuppressants during the first 6 months of treatment, as well as long-term therapy (103–107). Early administration within the first 36 vs. 72 months seemed to favor a protective value in visual outcome and disease control (108).

Similar to ADA, IFX is usually taken with another immunosuppressant drug to guard against anti-chimeric antibodies and might be associated with reactivation of tuberculosis and Hepatitis B or C diseases. Numerous adverse effects have been reported with IFX such as allergic reactions, induced lupus, aggravation of multiple sclerosis, optic neuritis and pulmonary embolism that might necessitate cessation of the drug (103, 104).

Several comparative studies between ADA and IFX have been conducted (109). Prominently, a multicenter study on 177 patients compared ADA with IFX as first line biologic in cases with refractory BD uveitis, and found that both groups had significantly better control in terms of disease activity but the ADA group had higher percentage of patients with better BCVA and higher drug retention rate with fewer drug related reactions (110).

Regarding the biosimilar IFX (bio-IFX), few contradicting reports exist as to its efficacy in the management of ocular BD. While bio-IFX was found to be disappointing in 3 patients with ocular and neuro-BD and resulted in recurrence of activity after switching from reference drug to biosimilars (111), another study reported the success of bio-IFX in achieving remission in 4 out of 6 patients with BD involving uveitis, nervous system, vascular and joint involvement (112).

**Golimumab** is another totally humanized anti-TNF alpha antibody that appears to have promising efficacy, notably in refractory BD cases (113, 114). Additional studies are necessary to better evaluate the efficacy and safety profile.

### Interferon alpha-2a

Interferon alpha is a cytokine produced in nature in response to a viral infection or tumor with variable antiviral, antiproliferative, antiangiogenic and immunomodulatory effects. In medical practice, interferon alpha-2a is generally indicated as second-line therapy in resistant cases, or as a first-line treatment in very severe posterior uveitis or in cases of intolerance to conventional immunosuppressive medications. Studies have revealed that it improved visual acuity, resolved macular edema, significantly reduced the rate of relapses, and sometimes allowed for steroids to be completely withdrawn (115, 116).

There is no standardized consensus regarding initial dosing up to reaching the maintenance dose, fulfilling remission and quiescence for a minimum of 6 to 9 months. However, upon commencement of therapy, oral steroids should be lowered to a maintenance dose of 10 mg/day (117, 118). The main side effects of interferon are a flu-like syndrome, psoriasis, epilepsy, depression, leukopenia and autoimmune manifestations (119).

### Interleukin-6 (IL-6) antagonists

#### Tocilizumab (TCZ)

During the past few years, there has been several reports demonstrating the efficacy of TCZ, an interleukin-6 inhibitor, in the control of BD uveitis cases refractory to conventional treatment and TNF-alpha blockers (120–122). The drug was able to achieve complete remission in some of the ocular Behçet cases, although it was not successful in systemic control of the disease in the same patients (123, 124) and may be considered in selected patients with refractory uveitic macular oedema (STOP-Uveitis Study) (125). The SATURN and SARIL-NIU trials focused on sarilumab, a newer IL-6 antagonist, in non-infectious uveitis. However, sarilumab has not yet been established in managing BU (126).

### Interleukin-1 (IL-1) antagonists

#### Anakinra (ANA) and canakinumab (CAN)

Both ANA & CAN are currently under investigation in the treatment of BU. A retrospective Italian multicentric study in 2017 stated these two IL-1 antagonists were successful in managing intraocular inflammation in a small cohort of Behçet patients (127, 128), a result further endorsed in another study, that reports a better BD patient response to IL-1 therapy in those with BD uveitis vs. BD without ocular involvement (129). The rationale for IL-1 inhibition and its reported success is based on the possible role played by IL-1β expressed by retinal dendritic cells, macrophages and neutrophils as a mediator of the local inflammatory process (130).

### Interleukin-17A (IL-17A) antagonists

The SHIELD trial was conducted to assess the efficacy of secukinumab in BD uveitis. The trial failed to meet its primary objective vs. placebo in uveitis recurrences, however, it significantly reduced the requirement for concomitant immunosuppressive treatment (125).

### Janus kinase inhibitors (JAKi)

Several studies have recently reported success with JAK inhibitors in the treatment of non-infectious autoimmune uveitis refractory to conventional DMARDs and anti-TNFα agents, suggesting they could be an alternative to the aforementioned (131, 132). Some have also reported steroid-sparing success. JAKi have already been approved in several rheumatological, gastrointestinal and dermatological autoimmune diseases. They act by inhibiting JAK-transmembrane protein phosphorylation, thus blocking or downregulating the cytokine expression cascade prior to its initiation (133). Zou et al. report successful results with tofacitinib BD patients with refractory BD uveitis, meriting a larger prospective controlled trial (133).

## Moving the systemic to the local environment

Given their systemic success and the booming era of anti-VEGF drugs, it was inevitable, that trials would soon follow, testing anti-VEGFs on one hand (in controlling the CME element of the inflammation), but more prominently the introduction of intravitreal injections of Infliximab initially, followed by Adalimumab (134–138). The rationale was to concentrate the treatment on site as well as to evade systemic side effects (134).

While Hamza et al. considered IFX IV injections a potential and safe, yet temporary option to consider for Behçet posterior uveitis with its drawback being a short study design of 18 weeks duration (138). A recent Egyptian study assessed the efficacy of 9 doses of monthly intravitreal IFX as an adjunct to systemic treatment, in 22 eyes of 16 patients with active posterior uveitis. Only 7 eyes achieved success (35%), in the remaining 13 (65%) failure was due to inability to control the inflammation or due to severe flaring of inflammation. The authors concluded that IV IFX for active posterior uveitis in Behçet's disease was associated with a high complication rate, failure to control inflammation in most eyes and could not be considered a substitute to systemic therapy (139).

In conclusion, so far studies are small and results remain inconclusive, while the desired favorable outcome seemed only temporary. Safety profiles, the issue of possible acquired immunogenicity, need for repeated injections and open questions regarding clinical benefit and quality of life remain topics for more extensive research (134).

## Behçet's uveitis and Covid-19

BD patients may be candidates for immunosuppression and hence more liable to contract serious infections compared to healthy individuals. A fine, critical balance is needed in BD patients with Covid-19 in an attempt to decrease mortality from the infection as well as avoid disease activity relapse. According to current expert recommendations, there is no reason to discontinue topical treatments, colchicine, and non-steroidal anti-inflammatory drugs. There may be a rationale to consider lowering systemic steroids to the lowest possible dose necessary. In cases with COVID-19 symptoms, immunosuppressive and biological agents can be temporarily stopped, but the decision should be tailored according to the patients' needs. Considering their potential beneficial effects on the course of COVID-19; colchicine, pentoxifylline, and dapsone can be considered as safe treatment options where indicated in BD. However, their role needs further evaluation (140). A retrospective analysis conducted by Bolletta et al. showed that despite immunosuppression (or some patients having stopped treatment) along with Covid-19 infection in Behçet patients, few of their cohort required hospitalization, none was admitted to the ICU and eventually about one third had exacerbation in at least one of their BD-related symptoms (141).

Although BD patients are recommended to receive SARS-CoV-2 vaccine, there have been reports of post-vaccination emergence or reactivation of BD and possible ocular inflammatory flare ups (142, 143).

## Conclusion

BD maintains a somewhat elusive nature to clinicians due to its heterogeneous presentations and its mimicry of other inflammatory diseases, as well as its ability to progress rapidly—and sometimes unexpectedly. This is mirrored in the multitude of classifications constantly developed and modified in an attempt to truly define this disease. A new tool expected to aid in classification, defining and identifying epidemiology, demographics, microbiome and genetic profiles of BD, and management data through real-life data collection are international and national registry programs, such as the AIDA Registry for BD. Management of BD and uveitis have seen a plethora of updates, especially pertaining to medical treatment and the entry of new investigative tools to aid in diagnosis, prognosis as well as disease monitoring and therapeutic response. The target remains to rapidly control the ocular inflammation and reduce the frequency and severity of relapses utilizing a combination of conventional therapies as well as the more recently biologic agents as defined by the latest EULAR guidelines.

## Author contributions

SA, MS, and GR: conceptualization, critical revision, and editing of the article. SA, RA, RE, LH, and MR: writing original draft. All authors reviewed and agreed on the final version.

## Conflict of interest

The authors declare that the research was conducted in the absence of any commercial or financial relationships that could be construed as a potential conflict of interest.

## Publisher's note

All claims expressed in this article are solely those of the authors and do not necessarily represent those of their affiliated organizations, or those of the publisher, the editors and the reviewers. Any product that may be evaluated in this article, or claim that may be made by its manufacturer, is not guaranteed or endorsed by the publisher.
